# Consider the Risk of Vesicouterine Fistula in the Event of Intermittent Fluid Vaginal Discharge after a Cesarean Section

**DOI:** 10.3389/fsurg.2017.00058

**Published:** 2017-10-17

**Authors:** Pascal Talla, Maria Ekotomati, Yves Brünisholz, Jean Bouquet de la Jolinière, Bernice Fagan, Anis Feki, Nordine Ben Ali

**Affiliations:** ^1^Department of Gynecology and Obstetrics, Neuchâtel Cantonal Hospital, Fribourg, Switzerland; ^2^Department of Gynecology and Obstetrics, Fribourg Cantonal Hospital, Fribourg, Switzerland

**Keywords:** caesarean section, vesicouterine fistula, vaginal discharge, caesarean complication, bladder lesion

## Abstract

Though the number of Vesicouterine Fistulas is steadily increasing in association with the increasing number of cesarean sections, clinical presentation can be subtle and not easily identified. In this case study, we will review the presenting symptoms of this complication and suggest an updated management protocol.

## Introduction

Vesicouterine fistula (VUF) represents 2–9% of all urogenital fistulae. The increasing rate of cesarean sections is associated with a growing number of cases reported in medical literature. In developed countries, most cases occur as a complication of Cesarean section, whereas in developing countries, VUF is mainly a complication following prolonged labor or vaginal obstetric procedures (1).

Youssef first described “Menouria” secondary to a VUF complicating a cesarean extraction in 1957. Menstruation is evacuated through the bladder *via* the VUF (may even cause amenorrhea). The flow can also be inverted, with urine leaking through the cervix into the vagina imitating urinary incontinence or causing persistent unexplained vaginal discharge following a cesarean section.

In 1999, Maciej Jozwik introduced a classification of VUF based on the routes of menstrual flow:

Type I: amenorrhea (absence of vaginal menstrual bleeding), menouria, and absence of urine leakage (the so-called Youssef syndrome),
Type II: menouria, normal menses, and intermittent urine leakage and
Type III: absence of menouria, normal menses, and intermittent urine leakage.

We will present the case of a patient with prolonged and intermittent vaginal discharge resulting from a VUF following a cesarean section. Note the prolonged delay of 6 months before to establish the diagnosis.

## Case Report

The patient has given written consent for the publication of this case study.

A 30-year-old primigravida woman in labor was admitted to the labor ward at term.

The presenting fetal pole failed to descend into the pelvis after 2 h of full cervical dilation and the fetal heart rate monitoring was deemed pathological. An emergency cesarean section was performed according to the “Misgav Lagash technique.” The surgeons encountered no difficulties during the operation and the patient was discharged home on the fourth post operatory day without complications.

One month after the operation, the patient consulted her physician for intermittent vaginal discharge. The vaginal examination was normal: The vaginal mucosa was not irritated; there was a limited presence of vaginal fluid with normal wet mount microscopy. An empirical local antiseptic treatment was prescribed.

The patient continued to report persistent intermittent vaginal discharge. She was referred to our department 6 months after the cesarean section. Upon questioning, she specified that the vaginal discharge was present every day and increased after micturition. The amount of the liquid discharge required one to two sanitary towels per day.

A tampon test (the bladder is filled with methylene blue dye after vaginal tampon insertion, transfer of the dye to the tampon proves the presence of a fistula) confirmed the diagnosis of a urogenital fistula.

**Figure 1:**
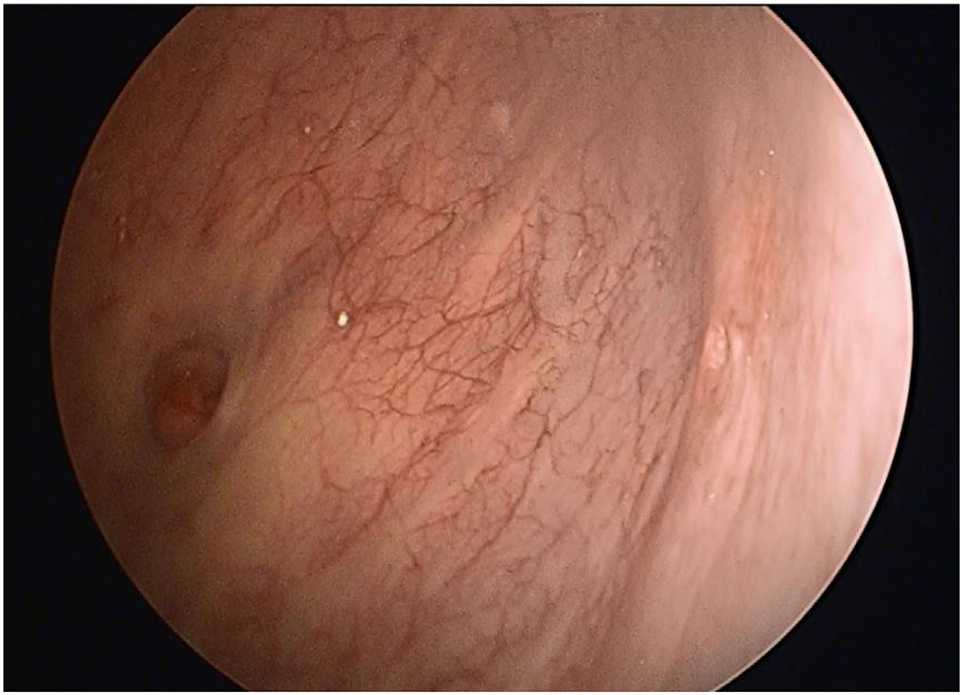
Cystoscopic view of the vesicouterine fistula on the right and the left ureteral ostium on the left.

**Figure 2:**
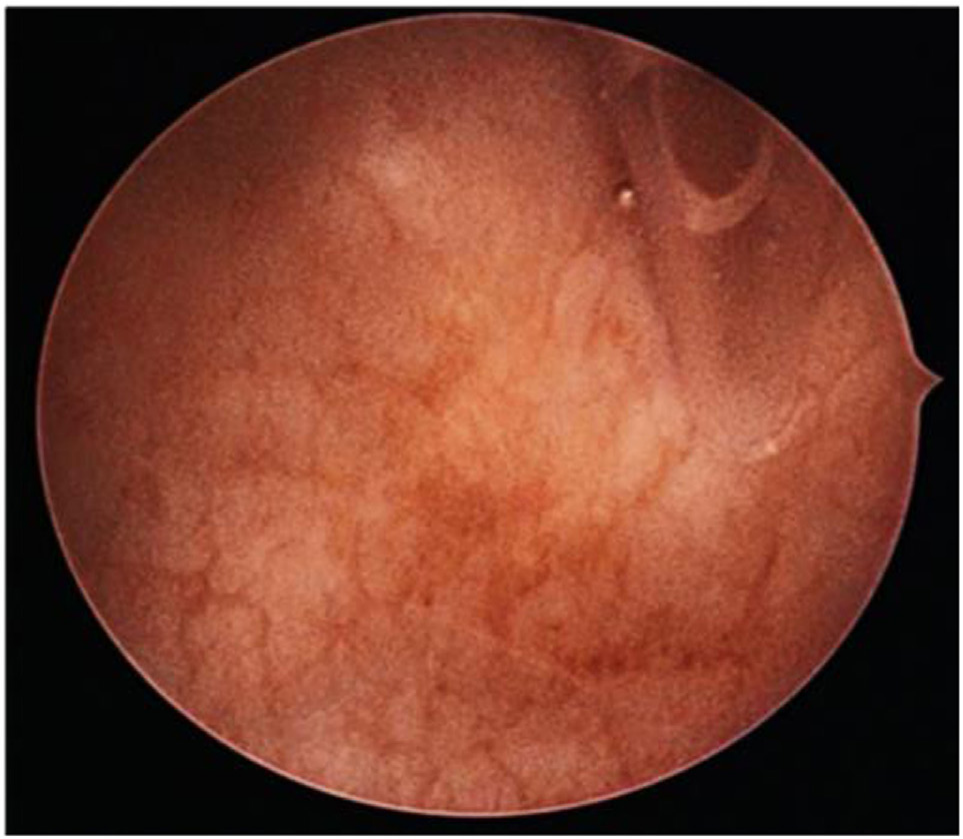
Hysteroscopic view of the urinary catheter introduced through the fistula in the bladder.

We were able to locate the fistula using combined cystoscopy and hysteroscopy (Figures 1 and 2).

After discussion, the patient chose to have the fistula surgically repaired. The surgeon selected a laparotomy access *via* Pfannenstiel to approach the fistula site.

The bladder was completely separated from the uterine scar and the fistula tissue excised. The bladder was closed in two layers with a running suture using PDS 2.O. The uterine wall was closed with a polyglactin1 X-stitch. An omental flap was placed between the vesical and uterine sutures to obliterate dead space and fixed.

An indwelling urinary catheter was left in place for 10 days.

The 3-month postoperative cystoscopy showed a normal vesical mucosa and the tampon test was negative.

One year later, the patient gave birth to her second child by a programmed cesarean section at term. The omentum was easily separated from its vesicouterine adhesion and the bladder was easily manipulated to enable a large exposure of the lower uterine segment. The operation was without complications, and the long-term follow-up was without fistula recurrence.

## Discussion

Urogenital fistula is an abnormal communication between the vesical bladder and the female genital tract. In developed countries, most of the cases occur as a complication of cesarean section or other gynecological operations and, less often, after obstetrical complications or different pelvic diseases or irradiation (2–4). In developing countries, urogenital fistulae occur mostly after a prolonged obstructed labor (1).

The World Health organization estimates 130,000 new cases of urogenital fistula occur per year. VUF represent only 2–9% of all urogenital fistulae (5, 6). Described for the first time in 1935, their prevalence was rare until 1947. Since then, their number has increased following the increasing number of cesarean sections and vaginal births following a previous caesarian section (7). A thin lower uterine segment and the presence of scar tissue between the uterus and the bladder related to a previous cesarean section may lead to a VUF as shearing forces are transmitted during labor.

An inadequate reflection of the bladder from the lower uterine segment during cesarean section is the main cause of injury of the bladder either during uterotomy or uterine closure. The devitalization of the posterior wall of the bladder with the presence of hematoma or infection could be the reason for a delayed presentation of the fistula (8).

Other less frequent causes of VUF reported in literature are the manual removal of the placenta, abnormal implantation of the placenta, uterine perforation, and migration of intrauterine device (9) cervical cerclage (10), endometriosis, uterine arteries embolization, necrobiosis of uterine fibroids, inflammatory bowel disease, and pelvic irradiation (11, 12).

A particularity of this type of fistula is that urinary leakage is not always present (see VUF classification in the introduction). Patients with a fistula situated above the internal os of a competent cervix, with the intrauterine pressure higher than intravesical, are continent (13). The VUF often manifests with cyclic hematuria (termed menouria by Youssef) as menses leak into the bladder through the fistulous track. The result is called “Yousseff Syndrome” described for the first time in 1957 and is associated with amenorrhea (absence of vaginal menstrual bleeding), cyclic menouria, and absence of urinary leakage.

The intermittent urine leakage can be explained by the presence of small fistulae, which are permeable during certain phases of the menstrual cycle.

Other presenting complaints can include perineal irritation, vaginal fungal infections, and recurrent urinary tract infections. Infertility and first trimester abortion are also associated. The diagnostic procedure of choice is the tampon test in cases of clinical suspicion of urine leakage through the uterine cervix. The instillation of methylene blue dye or indigo carmine is easily done with limited discomfort. Cystoscopy is usually performed to determine the size and location of fistula tract and its relation with the ureteral meatus (14).

Other imaging procedures can be useful to fully characterize the fistulous tract. There is no clear consensus on the best modality (15) and often more than one technique is required (16). Cystography, hysterography, excretory urography but also ultrasonography with or without intrauterine saline infusion (17, 18) can complete the diagnosis workup. False negatives can occur when sciagraphic pressure is not sufficient to cross the fistulous orifice (19). Helical computer tomography and MRI can be helpful for a patient when several other tests were inconclusive but should not be used as a first line imaging.

Conservative management by the placement of a bladder catheter for 4–6 weeks has been reported for small early diagnosed fistulae (<6 weeks from the causative surgery) (20) with a complete recovery in 5% of the cases. Simultaneous hormonal manipulation with induction of amenorrhea seems to promote spontaneous healing, as histological examination of fistulous tracts showed epithelial and stroma cells that contain sex hormone receptors as in the endometrium (21). There is no consensus for the length of the treatment, but most authors propose a duration of 6 months. Endoscopic fulguration of the fistula with induction of amenorrhea has also been reported (22, 23).

For most patients, VUF surgery is the definitive treatment. Upon the optimal timing of corrective surgery, some authors await the complete uterine involution as well as tissue healing (24). For fistulae post cesarean section, delaying the operation by up to 3 months seems to be preferred when possible. Different approaches have been reported for surgical closure of VUF including vaginal, extra peritoneal or trans peritoneal laparotomies, laparoscopic and robotic procedures (25–27).

The surgical technique usually used is based on the O’Connor’s technique applied for the treatment of VUF. The bladder is mobilized with the dissection of the fistulous tract and the opening of the uterus cavity. The bladder is repaired with two layers of stitches and the uterus is closed with a one layer suture. A vascularized tissue interposition like an omental flap is usually placed to obliterate dead space and prevent hematoma formation (28). The use of a parietal peritoneum flap has also been reported (28).

Vaginal approach is mostly used for VUF when an adequate mobilization is not possible for fistulae situated higher in the posterior wall of the bladder (29, 30). Hysterectomy can also be offered to patients with no desire to keep their uterus.

Concerning the future fertility after the correction of the VUF, Rajamaheswari and Chhikara report a spontaneous pregnancy for every seven patients who had not completed their family (29).

## Conclusion

Vesicouterine fistula is a rare type of urogenital fistula. The major risk factor is a previous cesarean section, which explains the growing number of reported cases in literature. Clinical manifestations vary and can be discrete and misleading. The diagnosis of VUF should be considered when there is a history of previous cesarean section. The vaginal tampon dye test is an easy diagnostic test. Treatment of VUF is mostly surgical with the surgical approach depending on the skills of the surgeon. The limited results are reassuring with no apparent effect on patient’s future fertility. An elective cesarean section is preferred after surgical VUF repair.

## Ethics Statement

Clinical ethics committee of the Cantonal Hospital of Fribourg. Written consent procedure.

## Author Contributions

PT: operator of the surgery. ME: review of the literature. NA: redaction of the article, affiliation to the HFR hospital. JJ, YB, and AF: lecture and correction. BF: correction of the English language redaction.

## Conflict of Interest Statement

The authors declare that the research was conducted in the absence of any commercial or financial relationships that could be construed as a potential conflict of interest.
